# Downregulated miR-31 level associates with poor prognosis of gastric cancer and its restoration suppresses tumor cell malignant phenotypes by inhibiting E2F2

**DOI:** 10.18632/oncotarget.9288

**Published:** 2016-05-11

**Authors:** Huaidong Wang, Xiaotian Zhang, Yuxin Liu, Zhaohui Ni, Yan Lin, Zipeng Duan, Yue Shi, Guoqing Wang, Fan Li

**Affiliations:** ^1^ Department of Pathogenobiology, The Key Laboratory of Zoonosis, Chinese Ministry of Education, College of Basic Medicine, Jilin University, Changchun, Jilin, China; ^2^ The Key Laboratory for Bionics Engineering, Ministry of Education, Jilin University, Changchun, Jilin, China

**Keywords:** gastric cancer, miRNA, miR-31, E2F2, biomarker

## Abstract

The miRNA microarray analysis showed that miR-31 was reduced in gastric cancer. This study further assessed miR-31 expression and role of miR-31 in gastric cancer tissues and cell lines. The data showed that miR-31 expression was down-regulated in 40 cases of gastric cancer tissues compared to the adjacent normal tissues, and low expression of miR-31 was associated with poor tumor differentiation, lymph node metastasis, advanced T stage and worse overall survival of gastric cancer patients. Ectopic expression of miR-31 reduced tumor cell viability, enhanced apoptosis, arrested tumor cells at G1 transition, and reduced tumor cell migration and invasion in SGC-7901 and MGC-803 gastric cell lines *in vitro*. Enforced expression of miR-31 also inhibited growth of engrafted tumors *in vivo*. Luciferase reporter assays and western blot revealed that E2F2 is the direct target of miR-31. E2F2 expression was upregulated in gastric cancer tissues, and inversely associated with miR-31 levels, while knockdown of E2F2 expression mimicked miR-31 anti-tumor activity in gastric cancer cells, but the ectopic expression of E2F2 rescued the miR-31-mediated inhibition in gastric cell lines. Taken together, these results demonstrated that miR-31 acts as a crucial tumor suppressive activity by inhibiting E2F2s expression. Thus, miR-31 might be a candidate therapeutic target for gastric cancer patients.

## INTRODUCTION

Gastric cancer is the fourth most prevalent cancer and the second most frequent cause of cancer-related mortalities in the world, accounting for more than 720,000 deaths annually [1, 2]. To date, more than 70% of gastric cancer occurs in the developing world [3] and gastric cancer new case and mortality were the highest in China according to the WHO world cancer report 2014. Although recent advancement in gastric cancer early detection, therapy, and prevention partly enhanced survival rate of early gastric cancer, Stage IV gastric cancer is still incurable with a very poor 5-year survival rate of approximately 4~5% [4]. The curative procedure of gastric cancer is still not satisfactory because the early gastric cancer was difficulty to discover in clinic [5]. Tumor cell-unlimited proliferation and strong invasive and metastasis ability are the main causes of high malignancy degree and worse overall survival. Therefore, identification of molecular aberrations that can predict tumor progression and survival rate might lead to creating a novel diagnostic means and thereby improving prognosis of gastric cancer.

Towards this end, microRNAs (miRNAs) are a class of endogenous small noncoding RNAs with 18 to 22 nucleotides in length and functionally, miRNA can post-transcriptionally silence protein translation or mRNA degradation through binding to 3′-untranslated region (3′UTR) of the target mRNA [6]. Increasing evidence indicates that miRNA play does a vital role in tumor initiation, progression and metastasis, which accomplished through regulation of cell proliferation, apoptosis, cell-cycle, differentiation, invasion and migration [7, 8]. Our previous miRNA microarray study showed that miR-31 was down-expressed in gastric cancer tissues [9]. Recently, a growing body of evidence indicates that miR-31 was involved with tumor progression and unfavorable prognosis in a variety of cancers, such as hepatocellular carcinoma [10], breast cancer [11], bladder cancer [12], lung cancer [13]. In gastric cancer, miR-31 was significantly down-regulated, but there was limited study showing whether miR-31 could influence tumor progression in gastric cancer. Hence, analysis of miR- 31 expression during tumor progression and metastasis may provide an innovative strategy for diagnostic and treatment of gastric cancer. In this study, we analyzed miR-31 expression in gastric cancer tissue samples and cell lines and then assessed the effects of miR-31 expression in gastric cancer cells *in vitro* and *in vivo*. As we know, miRNA is through inhibition of the targeting gene expression; thus, we explored and identified the targeting gene of miR-31 via bioinformatical analysis. We also analyzed expression of the target gene in gastric cancer tissues and cells and knocked down expression of the target gene and its role in gastric cancer cells.

## RESULTS

### Down-regulation of miR-31 expression in gastric cancer tissues and cell lines

**Figure 1:**
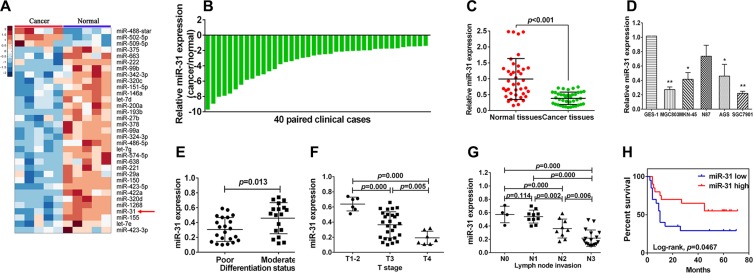
Downregulation of miR-31 expression in gastric cancer cell lines and tissues (**A**) Hierarchical cluster heat map of differentially expressed miRNAs in gastric cancer and corresponding normal tissues from microarray data. Yellow arrow denotes miR-31. (**B**) qRT-PCR analysis of miR-31 expression in 40 pairs of gastric cancer and corresponding normal tissues. miR-31 expression was normalized to U6. (**C**) Downregulation of miR-31expression in gastric cancer. (**D**) miR-31expression in gastric cell lines (GES-1, MGC-803, MKN-45, N87, AGS and SGC-7901) compared with normal gastric epithelial cells GES-1 detected using qRT-PCR. *p < 0.05 and **p < 0.01. (**E**) miR-31expression in different differentiation status of gastric cancer tissues (poorly differentiated = 22, moderately differentiated = 18). (**F**) miR-31expression in different tumor T stages (depth of cancer invasion), including 6 cases of T1–2 (mucous and muscular layer), 27 cases of T3 (serosal layer), and 7 cases of T4 (whole layer). (**G**) miR-31expression in different N stage (lymph node metastases) of gastric cancer (N0 = 4, N1 = 10, N2 = 9, N4 = 17). (**H**) Kaplan-Meier curve of overall survival of gastric cancer patients with high (*n* = 20) vs. low (*n* = 20) miR-31 levels (*p = 0.046*).

Our previous microarray miRNA data showed altered miR-31 expression in gastric cancer tissues compared to the corresponding normal tissues (Figure 1A). In this study, we then further assessed miR- 31 level in 40 gastric cancer and adjacent normal tissue samples using qRT-PCR (Figure 1B). Our data showed that miR- 31 expression was significantly lower in gastric cancer than in adjacent normal tissues (Figure 1C, *p* < 0.001). Furthermore, we also assessed miR-31 level in gastric cancer cell lines (MGC-803, MKN-45, AGS, and SGC- 7901) and normal gastric epithelial cells GES- 1 and found reduced miR-31 expression in gastric cancer cell lines (Figure 1D, *p* < 0.05), but have no statistical significance in N87 cell lines.

### Down-regulation of miR-31 expression associates with clinicopathological characteristics and prognosis of gastric cancer patients

We then associated miR-31 expression with clinicopathological data (including age, gender, tumor size, differentiation status, lymph node invasion, T stage, and distant metastasis). We divided patients into high and low expression of miR-31 according to the median level of miR-31 expression. We found that a remarkably low miR-31 level was significantly associated with poor tumor differentiation (*p* < 0.05), lymph node metastasis (*p* < 0.05), and advanced T stage (*P* < 0.05; Figure 1E–1G). However, there was no significant association of miR- 31 expression with age, gender, tumor size, and distant metastasis. Moreover, Kaplan-Meier analysis indicated that patients of miR-31 low expressed tumor tended to have worse overall survival than those with high miR-31 expressers (*p* = 0.046, Figure 1H).

### miR-31 restoration functionally suppresses proliferation, induces apoptosis and blocks G1 transition in gastric cells

**Figure 2:**
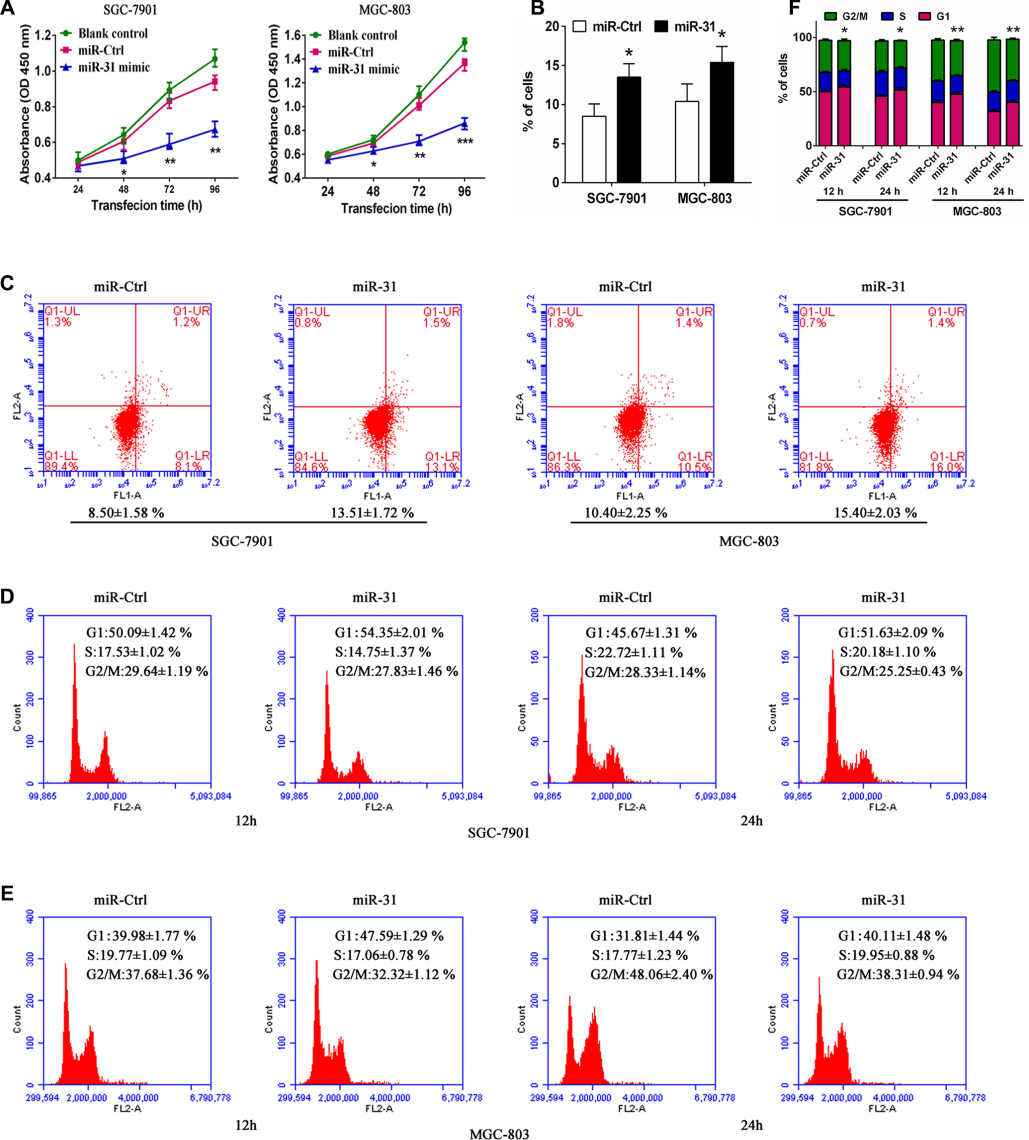
Ectopic expression of miR-31 inhibited tumor cell viability and induced apoptosis and cell-cycle arrest at theG1 phase in SGC-7901 and MGC-803 cells (**A**) Cell morphology. Tumor cells were transiently transfected with miRNA negative control ormiR-31 mimic for up to 96 h. (**B**) Cell viability CCK8 assay. The duplicated cells were then subjected to cell viability CCK8 assay. Data were presented as mean ± sd of three independent experiments. *p < 0.05, **p < 0.01 and ***p < 0.001. (**C**) Apoptosis assay. 48 h after transfection, tumor cell apoptosis was assessed to determine rate of early apoptosis of SGC-7901 and MGC-803 cells. Data were presented as mean ± sd of three independent experiments of duplicated samples. *p < 0.05 and **p < 0.01. (**D**, **E**, **F**) Flow cytometric cell cycle distribution assay. Additional culture of 12 and 24 h after 48 h transfection, cells were subjected to flow cytometric analysis of cell cycle distribution in SGC-7901 and MGC-803 cells. Experiments were repeated at least three times with similar results and the data are expressed as mean ± sd. *p < 0.05 and **p < 0.01.

Next, we assessed the effects of miR-31 restoration on regulation of gastric cancer cell proliferation, apoptosis, and cell cycle distribution. We transfected miR-31 mimic or miRNA negative control into two human gastric cancer SGC-7901 and MGC-803 cell lines, which have relatively lower levels of miR-31 expression to restore miR-31 expression. As expected, ectopic miR-31 expression was markedly suppressed SGC-7901 and MGC-803 cell proliferation (*p* < 0.05, Figure 2A). Furthermore, overexpression of miR-31 also induced apoptosis of both SGC-7901 and MGC- 803 cellsafter 48 h transfection (*p* < 0.05, Figure 2B, 2C). In addition, miR- 31 expression also arrested tumor cell at G1 phase of the cell cycle and decreased the proportion of cells at S phase and G2/M phase after 12 and 24 h post transfection (Figure 2D–2F). These data suggest that miR- 31 effectively reduces cell viability and induced apoptosis of gastric cancer cells.

### miR-31 restoration reduces migration and invasion in gastric cancer cells

**Figure 3:**
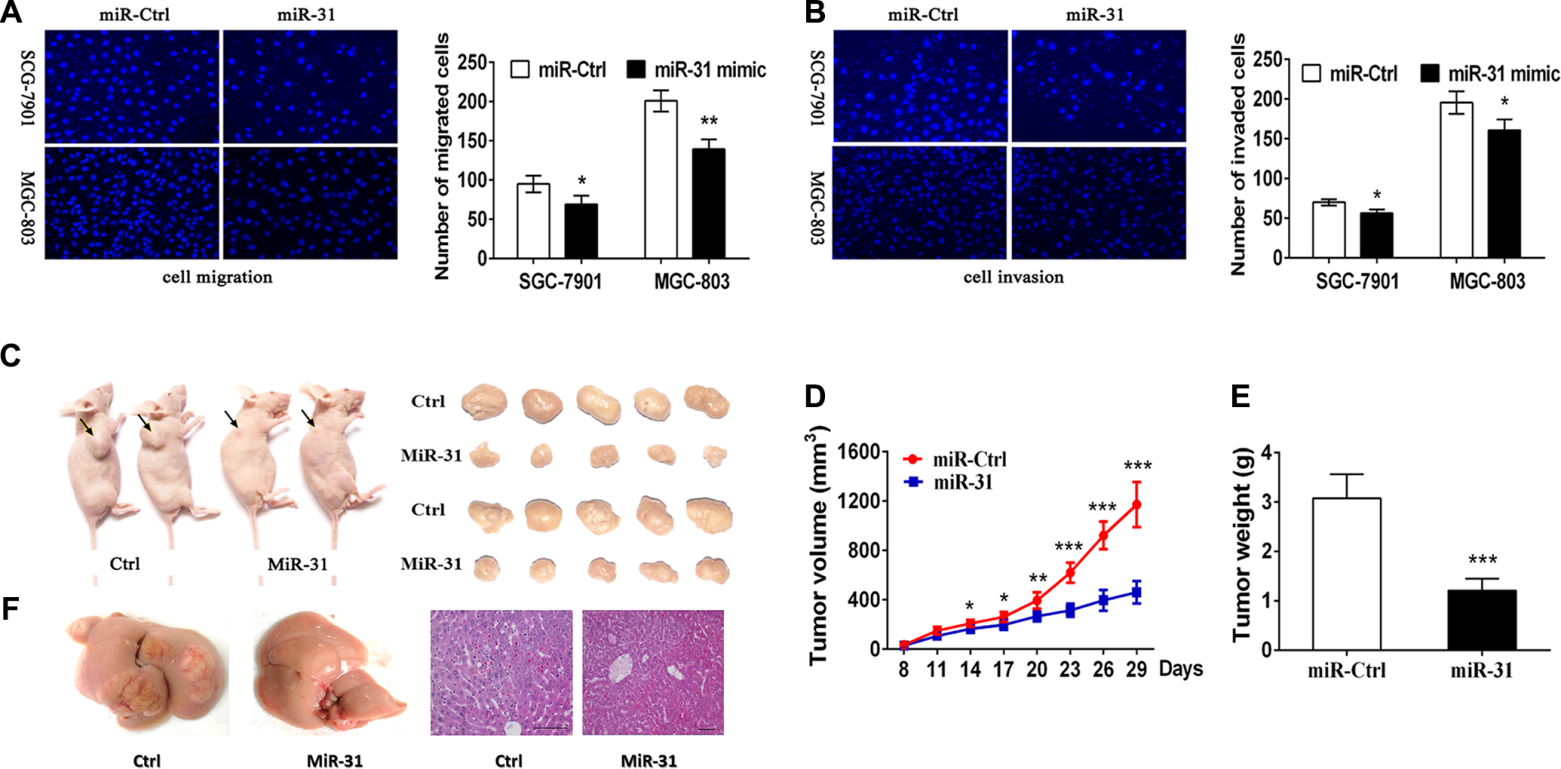
Suppression of gastric cancer cell migration and invasion and nude mouse xenograft formation and growth after restoration of miR-31 expression (**A**) Tumor cell Transwell migration assay. Fluorescent images (×200) (left) and quantification (right) of SGC-7901 and MGC-803 cell migration after 24 htransfection with miRNA negative control or miR-31 mimics. (**B**) Tumor cell Transwell invasion assay. Fluorescent images (left) and quantification (right) of SGC-7901 and MGC-803 cell invasion after 24 h transfection with miRNA negative control or miR-31 mimics. (**C**) Nude mouse xenograft assay. Subcutaneous tumor growth in mouse xenografts at 29 days after SGC-7901 cells were injection with miRNA negative control or miR-31 mimics. (**D**) Time-dependent tumor volumes (mm^3^) of miRNA negative control and miR-31 mimics mice. Tumor volume was measured at different time points and calculated with the following formula: tumor volume = length × width^2^ × 0.5. *p < 0.05, **p < 0.01, and ***p < 0.001. (**E**) Tumor weight formed by the indicated cells. ***p < 0.001. (**F**) Macroscopic observation and immunohistochemistry in the livers.

To further verify miR-31 function on the progression and metastasis of gastric cancer, we analyzed the effect of miR-31 overexpression on migratory and invasive capacity in gastric cancer cells. The results showed that miR-31 upregulation dramatically impaired the migration and invasion capacity of both SGC-7901 and MGC-803 cell lines (Figure 3A, 3B). These data suggest that miR-31 effectively inhibited tumor cell migration and invasion of gastric cancer cells *in vitro*.

### Restoration of miR-31 expression inhibits growth and metastasis of nude mouse xenografts

To evaluate the *in vivo* effects of miR-31 on gastric cancer tumor growth and metastasis, SGC-7901 cells were subcutaneously injected into the dorsal flank of nude mice with miR-31mimic or miRNA-control (Figure 3C). In accord with the tumor growth curve, both the volumes and weights of tumors formed by miR-31 mimics- transduced gastric cancer cells were lower and smaller than that of the corresponding control tumors (Figure 3D, 3E). In addition, SGC7901 cells stably expressing miR- 31 and miRNA-control cells were transplanted through the lateral tail vein to explore the effects of miR-31 expression on tumor metastasis. Macroscopic observation and histological analyses of their livers showed that the ectopic expression ofmiR-31 significantly inhibited metastasis in the organs (Figure 3F). These data indicate that miR-31 plays a pivotal role in gastric cancer progression *in vivo*.

### E2F2as the direct target of miR-31 in gastric cancer cells

**Figure 4:**
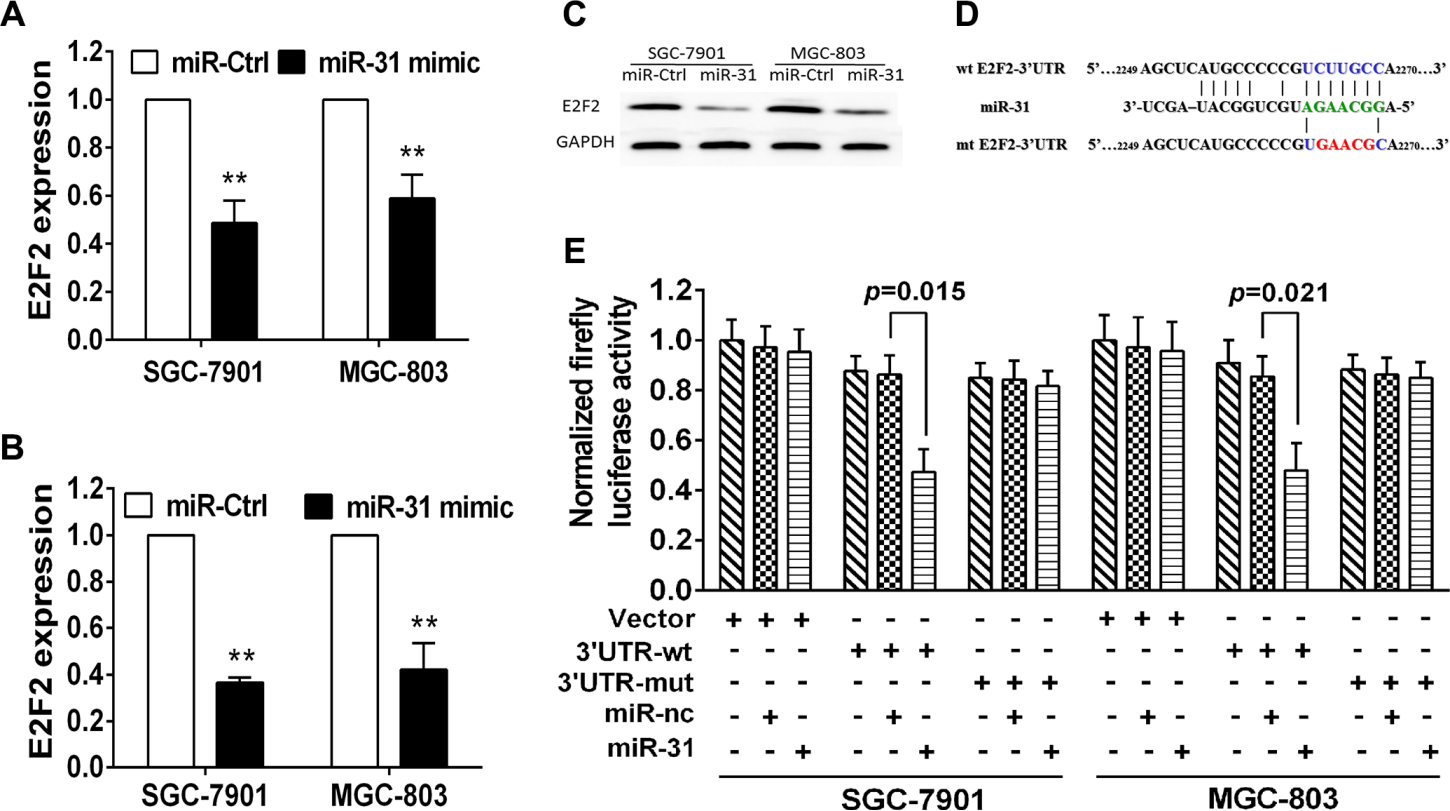
E2F2 is the direct target of miR-31 in gastric cancer cells (**A**) qRT-PCR. Level of E2F2 mRNA after miR-31 negative control or miR-31 mimic transfection into gastric cancer cells. (**B**, **C**) Western blot. Level of endogenous E2F2 protein after miRNA negative control or miR-31 mimic transfection into gastric cancer cells. The relative protein level was normalized to GAPDH and data were presented as mean ± sd. (**D**) Sequence alignment. miR-31 sequences and its predicted binding site in 3′-UTR of E2F2. Plasmids contain wild-type or mutant sequences. (**E**) Luciferase reporter assay. The vector containing wide-type E2F2 3′UTR or mutant E2F2 3′-UTR was co-transfected into SGC-7901 and MGC-803 cells together with miRNA negative control or miR-31 mimic transfection. Luciferase activity ratio was presented as firefly luciferase value/renilla luciferase value and then normalized to that of the empty plasmid. Each column represented mean ± sd.

To explore the underlying gene target of miR-31 in gastric cancer cells, we utilized the public bioinformatics tools (TargetScan, StarBase V2.0, microRNA.org, miRDB and miWalk) to search theoretical target genes and binding sites. We observedE2F2 may serve as the direct target of miR-31 in gastric cancer. We then performed RT-PCR and western blot analyses and found that transfection with miR-31 mimic significantly decreased level of E2F2 mRNA and protein in both MGC803 and SGC7901cells (Figure 4A–4C). Furthermore, bioinformatics analysis showed that 3′-untranslated region (3′-UTR) of E2F2 contained a conserved putative target site ofmiR-31 (Figure 4D). The luciferase reporter assay showed a significant reduction of luciferase activity of both SGC-7901 and MGC-803 cells aftermiR-31 mimic transfection compared to the negative control (Figure 4E). In addition, mutation of the predicted-binding site of miR- 31 on the E2F2 3′-UTR can rescue the luciferase activity (Figure 4E), indicating that E2F2 is a direct target of miR- 31 in gastric cancer cells.

### Upregulation of E2F2 expression in gastric cancer tissues and associates with clinicopathological characteristics and prognosis in gastric cancer patients

**Figure 5:**
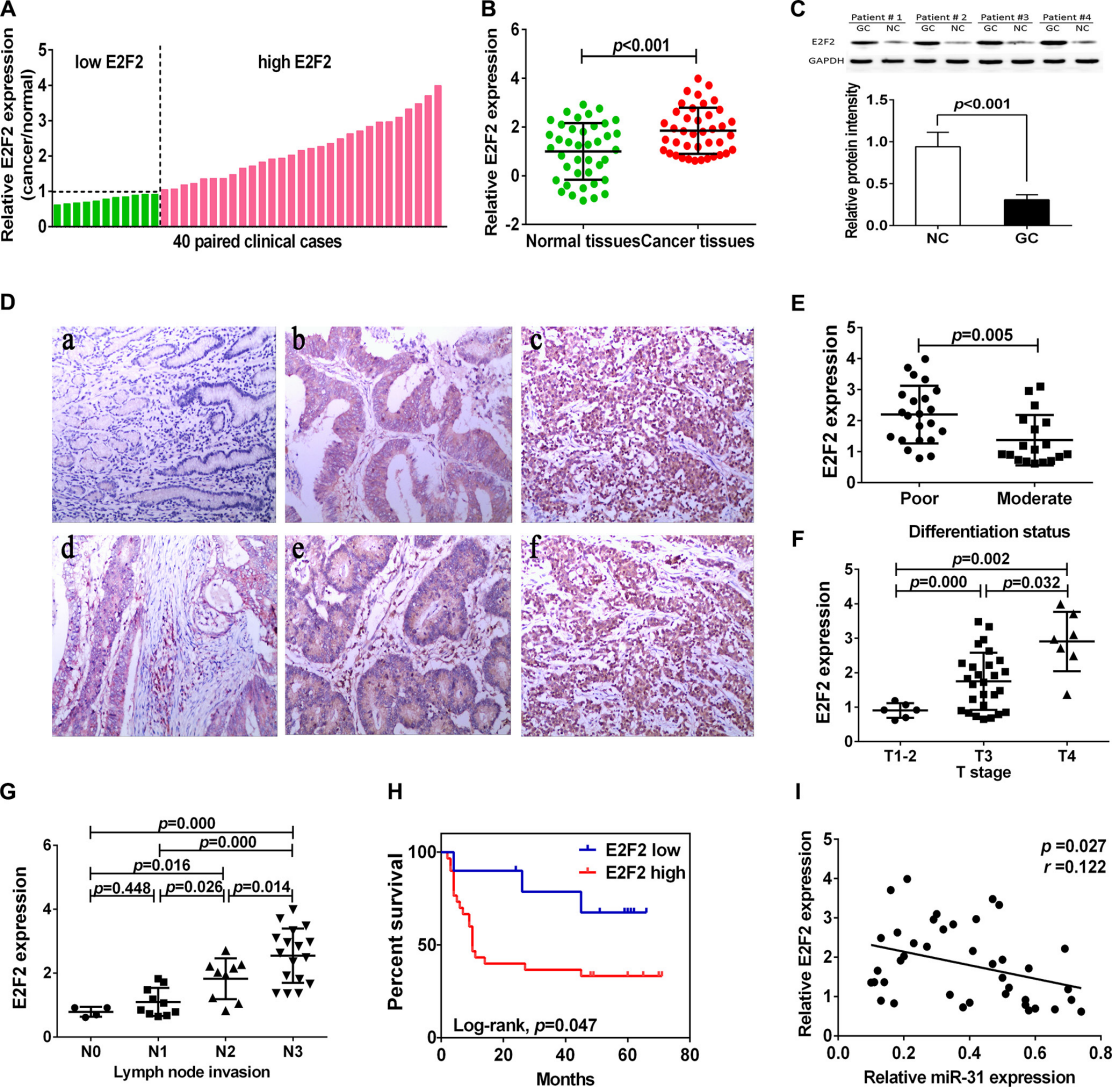
Upregulation of E2F2 expression in gastric cancer tissues (**A**, **B**) qRT-PCR analysis of E2F2 expression in 40 pairs of gastric cancer and corresponding normal tissues. Expression of E2F2 protein was normalized to that of β-actin. The level of E2F2mRNA was significantly higher than that of the adjacent tissues. (**C**) Western blot. Level of E2F2 protein was normalized to GAPDH. (**D**) Immunohistochemistry. E2F2 protein expression in gastric cancer tissues was analyzed by immunohistochemistry. a, normal gastric tissue; b, moderately differentiated gastric adenocarcinoma; c, poor differentiated gastric adenocarcinoma; d, superficial muscular infiltration; e, serosal layer infiltration; f, whole layer infiltration (all ×100). (**E**) Expression of E2F2 protein in different differentiation status of gastric cancer. (poorly differentiated = 22, moderately differentiated = 18). (**F**) Expression of E2F2 in different T stage (depth of cancer invasion) of gastric cancer, including T1–2 (mucous and muscular layer) 6 cases, T3 (serosal layer) 27 cases and T4 (whole layer) 7 cases. (**G**) Expression of E2F2 in different N stage (lymph node metastases) of gastric cancer (N0 = 4, N1 = 10, N2 = 9, N4 = 17). (**H**) Kaplan-Meier curve of overall survival of gastric cancer patients with high (*n* = 29) vs. low (*n* = 11) E2F2 levels (*p* = 0.047). (**I**) Comparison of miR-31 with E2F2 level in gastric cancer (*r* = 0.122, *p* = 0.027).

We then assessed E2F2 expression in gastric cancer tissue samples and found that level of E2F2 mRNA was increased in 29 of 40 gastric cancer tissues compared to the adjacent normal tissues (Figure 5A–5D). We then associated E2F2 expression with clinicopathological characteristics from 40 gastric cancer patients. Level of E2F2 expression was divided into high (*n* = 29) vs. low (*n* = 11) in these 40 patients. We found that a remarkably highE2F2level was significantly associated with poor tumor differentiation (*p* < 0.05), lymph node metastasis (*p* < 0.05), and advanced T stage (*p* < 0.05; Figure 5E–5G). However, there was no significant association of E2F2 expression with age, gender, tumor size, and distant metastasis. Moreover, Kaplan-Meier analysis indicated that patients with high E2F2-expressed gastric cancer tended to have worse overall survival than those with low E2F2-expressed tumor (*p* = 0.047, Figure 5H). Comparison of E2F2 mRNA expression with miR- 31in gastric cancer exhibited an inverse association (*r* = 0.122,*p* = 0.027; Figure 5I).

### Effects of miR-31 restoration on gastric cancer cells through inhibition of E2F2

**Figure 6:**
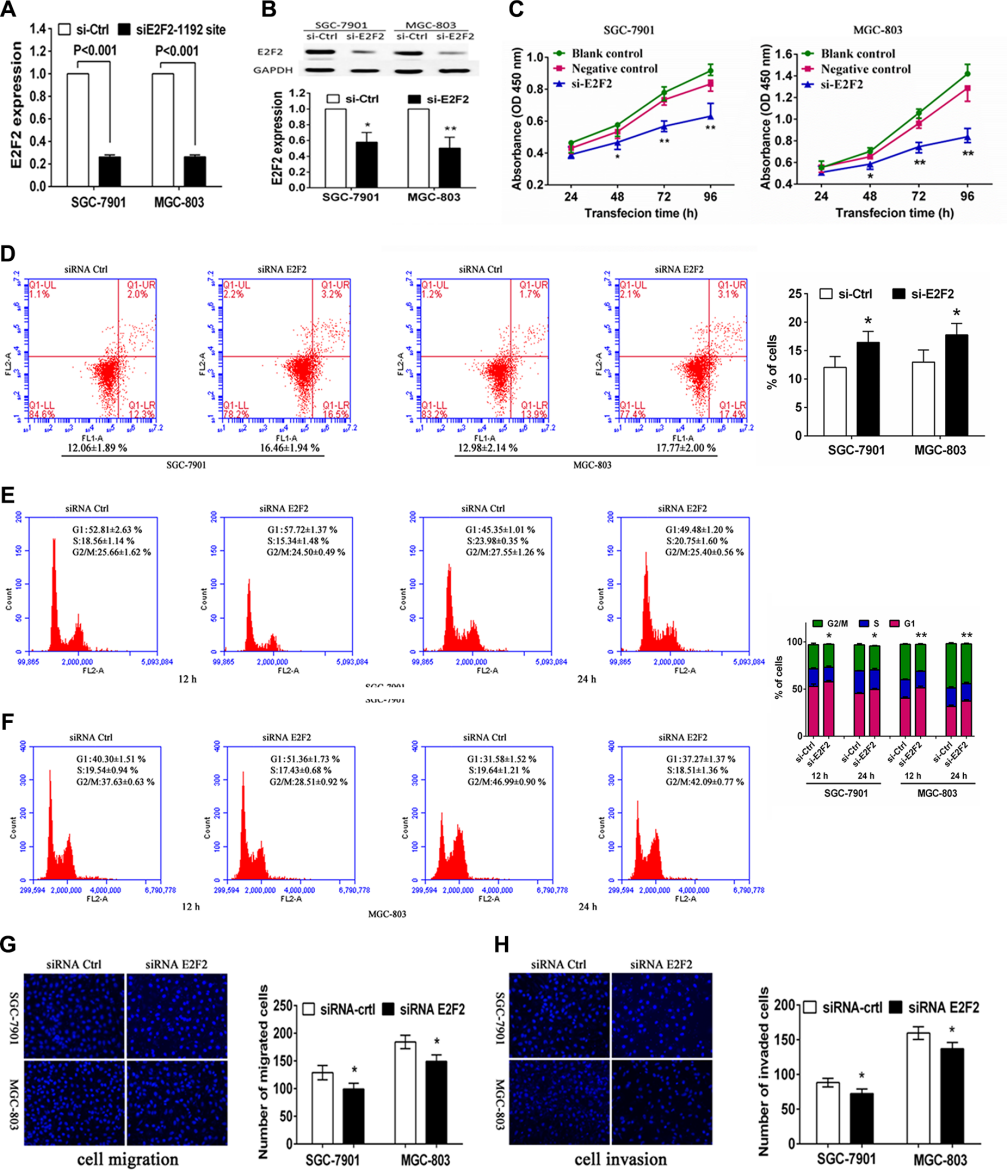
Effects of E2F2 knockdown on gastric cancer SGC-7901 and MGC-803 cells Tumor cells were transiently transfected with negative control siRNA orE2F2 siRNA and then subjected to different assays. (**A**) RT-PCR. Level of E2F2 mRNA was assessed by RT-PCR after transfection with negative control or E2F2 siRNA into gastric cancer cells. (**B**) Western blot. Level of E2F2 protein was assessed after transfection with negative control or E2F2 siRNA into gastric cancer cells. The relative protein levels were normalized to GAPDH and data were presented as mean ± sd. (**C**) Cell viability CCK8 assay. Cell viability was assessed after 24, 48, 72 and 96 h transfection with negative control or E2F2 siRNA into gastric cancer cells. Data were presented as mean ± sd of three independent experiments. *p < 0.05 and **p < 0.01. (**D**) Apoptosis assay. Level of apoptosis was assessed after 48 h transfection with negative control or E2F2 siRNA into gastric cancer cells. Data were presented as mean ± sd of three independent experiments with duplicated samples.*p < 0.05 and **p < 0.01. (**E**, **F**) Flow cytometric cell cycle assay. Cell cycle distribution was analyzed additional culture of 12 and 24 h after 48 h transfection with negative control or E2F2 siRNA into gastric cancer cells. Experiments were repeated at least three times with similar results and the data were expressed as mean ± sd. *p < 0.05 and **p < 0.01. (**G**) Tumor cell migration assay. Fluorescent images (×200) (left) and quantification (right) of migration level of SGC-7901 and MGC-803 cells after 24 h transfection with negative control or E2F2 siRNA. (**H**) Tumor cell invasion assay. Fluorescent images (left) and quantification (right) of invasion level of SGC-7901 and MGC-803 cells after 24 h transfection with negative control or E2F2 siRNA.

To evaluate whether E2F2 serves as a critical mediator of miR-31 in gastric cancer cells, we suppressed E2F2expression in SGC-7901 and MGC-803 cells using a specific siRNA (Figure 6A, 6B). Knockdown of E2F2 expression produced an anti-proliferative effect compared with siRNA control in both SGC-7901 and MGC-803 cells (Figure 6C). Moreover, transfection with E2F2 siRNA also significantly increased tumor cell apoptosis (Figure 6D), accompanied by a cell-cycle arrest at the G1 phase (Figure 6E, 6F). In addition, we found that knockdown of E2F2 reduced tumor cell migration and invasion capacity in these twoSGC-7901 and MGC-803 cell lines (Figure 6G, 6H). As expected, E2F2 siRNA transfection altered multiple biological behaviors that were similar to the results thatmiR-31was upregulated. In addition, the E2F2 expression vector pWSLV-01-E2F2 was used to restore E2F2 expression (Figure S1A, S1B). Overexpression of E2F2 rescued themiR-31-mediated inhibition of cell proliferation, cell cycle arrest, migration, invasion, and improve the cell apoptosis in SGC-7901 and MGC-803 cells (Figure S1C–S1H). These result suggested that E2F2 was the direct target of miR-31 in gastric cancer cells.

## DISCUSSION

MiRNA plays a regulatory role in inhibition of protein expression and altered expression of miRNAs was associated with cancer development and progression [14, 15]. In the current study, we found that miR-31 expression was down-regulated in gastric cancer tissues and cell lines, while down-regulation of miR-31 expression associates with clinicopathological characteristics and prognosis of gastric cancer patients. Moreover, miR-31 restoration reduced gastric cancer cell viability, migration and invasion capacity, but induced tumor cells to undergo apoptosis and arrested tumor cell at G1 phase of the cell cycle *in vitro*. Restoration of miR-31 expression inhibited growth of nude mouse xenografts *in vivo*. Mechanistically, miR-31 antitumor activity was through targeting of E2F2 expressionin gastric cancer cells.

MiR-31 is one of well identified miRNAs in cancer biology, and regulation patterns and functions of miR-31 were diverse depending on cancer types. It is hypothesis that miR-31 has a specific function in different tumor types was involved with methylation-dependent silencing and local deletion. Previous studies have shown that miR-31 acted as a potential tumor tumorigenesis in lung cancer [13], colorectal cancer [16] and head and neck carcinoma [17]. On the contrary to this, miR-31 acted as a potential tumor suppressor in other tumor types. It has been reported that miR-31 possessed multiple mechanisms to inhibit breast cancer metastasis by partial coordination to repress the metastasis-promoting genes, such as RhoA, ITGA5 and RDX [18]. The similar function of miR-31 has also been found in prostate cancer [19]. In another study, it identified that downregulation of miR-31 may disrupt normal cell homeostasis and contribute to evolution and progression of prostate cancer through promotion of androgen receptor signaling [20]. Furthermore, in ovarian carcinoma, miR31 inhibited proliferation of serous ovarian carcinoma cells by regulating the p53 pathways [21]. In the current study, we showed that miR- 31 was significantly downregulated in gastric cancer tissues compared with corresponding normal tissues. MIR31, which encodes p14^ARF^, are located at 9p21.3, a genomic region commonly deleted in many cancers, and E2F2overexpressionnormally leads via p14^ARF^, which may result in down-regulation of miR-31 in gastric cancer [21]. Downregulated miR-31 expression was significantly associated with poor tumor differentiation, lymph node metastasis, advanced T stage and worse overall survival, suggesting that miR-31 may have a suppressive role in progression of gastric cancer. Consistent with clinical data, further biological experiments showed miR-31 antitumor activities in gastric cancer SGC-7901 and MGC-803 cell lines *in vitro* and *in vivo*. However, since the miR- 31 could inhibit the cell's proliferation, but there was no significant association of miR-31 expression with tumor size of gastric patients. We speculated it was partly due to the unconspicuous miR-31 expression in intergroup difference or internal environment in patients. So the cell's proliferation which related to miR-31 expression *in vitro* research may not necessarily consist with tumor size in clinic data.

To gain a further insight into the mechanisms of miR-31 in gastric cancer, we screened the target genes of miR-31 by using public bioinformatics tools [22–24]. Eventually, E2F2 was identified as a candidate target of miR-31 for further investigation and our previous study also showed altered expression and role of E2F2 in gastric cancer [9]. Indeed, imbalance of cell-cycle was closely related to the malignant proliferation of tumor cell. E2F2 plays a vital role in regulating cell cycle progression [25]. Bioinformatics analysis suggested that E2F2 was identified as a direct target of miR-31 [21]. It has been reported that miR-31 suppressed E2F2 and triggered the p53-dependent apoptotic program to inhibit proliferation of serous ovarian carcinomas [21]. Another previous study of prostate cancer has suggested that E2F2 was a predicted direct target of miR-31 [20]. In the current study, we found that E2F2 expression was inversely associated with miR-31 levels in gastric cancer tissues. Level of E2F2 mRNA and protein was significantly reduced following the ectopic expression of miR-31 in gastric cancer cells. The luciferase activity assay further showed that E2F2 was a direct target of miR-31. Moreover, knockdown of E2F2 in gastric cancer cells produced an anti-proliferative, apoptosis increased, G1 phase retardation and migratory and invasive reduced effect, which was similar to the results when miR-31 upregulation. Moreover, the restoration of E2F2 expression in cells stably expressing miR-31 was able to counteract the inhibitory effects of miR-31 in the gastric cancer cells. Taken together, these data provide strong evidence that E2F2 is a direct and functional target of miR-31.

Moreover, our current results indicated that E2F2 was involved in progression, lymph node metastasis and prognosis of gastric cancer patients. E2F2 protein was a member of the E2F family, which plays a significant role in regulation of cell cycle process [26, 27]. Previous studies demonstrated that altered expression of E2F2 protein was closely associated with development of different cancers [25]. It has been reported that E2F2 mutation and its pathway activation were associated with tumor proliferation and survival of breast cancer patients [28, 29]. Another breast cancer study showed that loss of E2F2 expression significantly reduced tumor metastatic capacity after E2F2 reduction [30]. In hepatocellular carcinoma, E2F2 has been reported to be a tumor-promoter [31], while in prostate cancer, E2F2 also has been discovered inhibition of tumor cell proliferation through regulation of miRNA let-7a [32]. In the current study, we observed that E2F2 was overexpressed in gastric cancer tissues and moreover, high E2F2 levels were positively associated with poor tumor differentiation, lymph node metastasis, advanced T stage and worse overall survival. To verify the effect of E2F2 knockdown on progression of gastric cancer, we utilized specific RNAi to transfect it into gastric cancer cells and found that silence E2F2 could remarkably reduce cell proliferation, migration and invasion, but enhanced tumor cells to undergo apoptosis. These data demonstrated that knockdown of E2F2 expression had similar effects on gastric cancer to that of miR-31 overexpression.

In summary, our current study demonstrated that miR-31 possessed tumor suppressor effects in gastric cancer development and progression. Ectopic expression of miR-31 suppressed multiple malignant biological behaviors, including inhibition of tumor cell viability, enhancement of apoptosis, blockage of the G1 progression, and reduction of migration and invasion *in vitro* and tumor xenograft growth *in vivo*. Our further experiments revealed that E2F2 was a direct target of miR-31 in gastric cancer cells with evidence that E2F2 was overexpressed in gastric cancer, knockdown of E2F2 expression had similarly suppressive actions to miR-31 expression in gastric cancer cells, and E2F2 expression was inversely associated with miR-31 expression. Further study will confirm that miR-31 could be a potential therapeutic target for gastric cancer.

## MATERIALS AND METHODS

### Gastric tissue samples

In this study, we collected 40 case of tumor and distant normal tissue samples from gastric cancer patients between December 2008 and May 2011 from Jilin University (Changchun, China). This study was approved by the Ethics Committee of School of Basic Medical Sciences, Jilin University and each patient was consented in a written informed consent form. All patients were diagnosed with gastric cancer and underwent radical resections of gastric cancer. Their clinicopathological parameters, including age, gender, tumor size, differentiation status, lymph node metastasis, clinical stage, and distant metastasis, were collected from their medical records and summarized in Table 1. All tissues were obtained from the operation room and stored in liquid nitrogen within 10 min.

**Table 1:** Correlations between miR-31 and E2F2 expressions and clinical characteristics in patients with gastric cancer

Characteristics	Total No.	miR-31 expression			E2F2 expression		
		Low	High	*P*-value	Low	High	*P*-value
		No. case (%)	No. case (%)		No. case (%)	No. case (%)	
**Age**				0.055			0.623
< 60	18	6 (30)	12 (60)		5 (45)	13 (45)	
≥ 60	22	14 (70)	8 (40)		6 (55)	16 (55)	
**Gender**				0.095			0.157
Male	25	15 (75)	10 (50)		5 (45)	20 (69)	
Female	15	5 (25)	10 (50)		6 (55)	9 (31)	
**Tumor size**				0.264			0.422
< 5 cm	19	11 (55)	8 (40)		6 (55)	13 (45)	
≥ 5 cm	21	9 (45)	12 (60)		5 (45)	16 (55)	
**Differentiation status**							
Moderate	18	5 (25)	13 (65)	0.012*	9 (82)	9 (31)	0.005*
Poor	22	15 (75)	7 (35)		2 (18)	20 (69)	
**Lymph node invasion**				0.000*			0.008*
N0	4	0 (0)	4 (20)		4 (36)	0 (0)	
N1	10	0 (0)	10 (50)		5 (45)	5 (17)	
N2	9	5 (25)	4 (20)		1 (9)	8 (28)	
N3	17	15 (75)	2 (10)		1 (9)	16 (55)	
**T stage**				0.005*			0.030*
T1	2	0 (0)	2 (10)		2 (18)	0 (0)	
T2	4	0 (0)	4 (20)		2 (18)	2 (7)	
T3	27	13 (65)	14 (70)		7 (64)	20 (69)	
T4	7	7 (35)	0 (0)		0 (0)	7 (24)	
**Distant metastasis**				0.366			0.275
M0	28	13 (65)	15 (75)		9 (82)	19 (66)	
M1	12	7 (35)	5 (25)		2 (18)	10 (34)	

### Cell lines and culture

Human gastric cancer MGC-803, MKN-45, N87, AGSand SGC-7901 cell lines and a normal gastric epithelial cell GES-1 line were obtained from American Type Culture Collection (Manassas, VA, USA) and maintained in RPMI 1640 (Invitrogen, Carlsbad, CA, USA) supplemented with 10% FBS (Invitrogen) at 37°C in a 5% CO_2_ atmosphere.

### RNA isolation and qRT-PCR

Total RNA was isolated from tissue and cell specimens using Trizol reagent (Invitrogen) and miRNA was then isolated using miRcute miRNA isolation kit (Tiangen, Beijing, China) according to the manufacturer's instructions. RNA concentration was then measured using the Epoch Multi-volume Spectrophotometer System (BioTek, Vermont, USA) and these RNA samples were reversely transcribed into cDNA using a PrimeScript RT reagent Kit (TaKaRa Otsu, Shiga, Japan) and miRcute miRNA Fist-Strand cDNA synthesis kit (Tiangen, Beijing, China). qPCR was then performed using SYBR Premix Ex Taq (TaKaRa) and miRcute miRNA qPCR detection kit (SYBR Green) (Tiangen). β-actin and U6 were used to normalize the level of miRNA and mRNA expression. The primer of has-miR-31 and U6 were purchased from Tiangen, has-miR-31 and U6 primers were AGGCAAGAUGCUGGCAUA GCUor5′-CGCTTCGGCAGCACATATACTA-3′ and 5′-CGCTTCA CGAATTTGCGTG TCA-3′ respectively, while β-Actin and E2F2 primers were 5′-CTGGAACGGTG AAGG TGACA-3′ and 5′-AAGGGACTTCCTGTAACAATGCA-3′ or5′-CGTCCCTG AGTTCCCAACC-3′ and 5′-GCGA AGTGTCATACCGAGTCTT-3′, respectively. qPCR was performed in ABI 7300 system and the data were analyzed using the 2^ΔΔCT^ method.

### Protein extraction and western blot

Total cellular protein was extracted form tissue samples and cell lines using Cell lysis buffer for western and IP (Beyotime, Shanghai, China). These protein samples (20 μg each) were subjected to sodium dodecyl sulfate-polyacrylamide gel electrophoresis (SDS-PAGE) and transferred onto PVDF membranes (Millipore, Bedford, MA, USA). The membranes were blocked in 5% skimmed milk and then incubated with primary and secondary antibodies respectively. The immunoblots were visualized using Gene gnome syngene bio imaging (Syngene, Cambridge, UK) and protein levels were normalized to GAPDH with fold changes. A primary anti-E2F2 antibody was obtained from Santa Cruz Biotechnology (#sc-632, Santa Cruz, CA, USA) and used to conduct western blot and immunohistochemistry, while a GAPDH antibody and secondary antibodies were purchased from Beyotime (Shanghai, China).

### Immunohistochemistry

Paraffin blocks from gastric cancer and normal tissues were sectioned into 4-μM-thick sections. For immunohistochemistry, these sections were immunostained for E2F2 protein using the Envision method and immunostained sections were evaluated according to intensity and extent of immunoreactivity according to a previous study.

### Gene transfection

miR-31 mimic, miRNA mimic control, siRNA against E2F2 and siRNA control were synthesized by Ribobio (Guangzhou, China) and transfected into gastric cancer cell lines using Lipofectamine RNAiMAX (Invitrogen) according to the manufacturer's instructions. The pWSLV-01-E2F2 plasmid was constructed and transfected into gastric cancer cell lines using Lipofectamine LTX (Invitrogen).

### Luciferase reporter assay

A wild-type 3′-UTR fragment of E2F2 cDNA was amplified by using PCR and cloned into *Xba*I and *Sac*I site of pmirGLO dual-luciferase miRNA target expression vector (Promega, Madison, WI, USA) and named as wt E2F2-3′-UTR. The mutant variant of E2F2 3′-UTR was generated based on wt E2F2-3′-UTR by mutating five nucleotides that potentially bind to miR-31 and named as mt E2F2-3′-UTR. These vectors (wt E2F2-3′-UTR or wt E2F2- 3′-UTR were together with miR-31 mimic or miR-ctrl) were transiently transfected into gastric cancer SGC-7901 and MGC-803 cells using Lipofectamine 2000 reagent (Invitrogen). Luciferase activity was then detected 48 h later using the Dual-Glo luciferase assay system (Promega) using Synergy H1 Multi-Mode Microplate Reader (BioTek, Vermont, USA). Luciferase activity ratios were presented as firefly luciferase values/renilla luciferase values, after normalized to the control plasmid.

### Cell proliferation, apoptosis and cell-cycle assay

After gene transfection, tumor cell viability was assessed using Cell Counting Kit-8 (CCK8) kit (Dojindo Molecular Technologies, Kumamoto, Japan) according to the manufacturer's protocol. Experiments were repeated at least three times with similar data.

Furthermore, apoptosis assay was also performed after 48 h transfection with miR-31 mimic or siRNA E2F2intoSGC-7901 and MGC-803 cells using Annexin V FITC/PI Apoptosis Detection Kit (Roche, Basel, Swiss) and Accuri C6 flow cytometer and Cell Quest Pro Software (BD Biosciences, San Jose, CA).

In addition, cell-cycle analysis were performed additional culture of 12 and 24 h after 48 h transfection with miR-31 mimic or siRNA E2F2 into SGC-7901 and MGC-803 cells using cell cycle detection kit (Keygen, Nanjing, China) and Accuri C6 flow cytometer (BD Biosciences).

### Tumor cell migration and invasion assay

Tumor cell migration and invasion capacity was assessed using Transwell chamber (Corning, Corning, NY, USA) in 24-well plates. In brief, SGC-7901 and MGC-803 cells were transfected with miR-31 mimic or siRNA E2F2 and 24 h later, cells was resuspended in a serum-free RPMI 1640 medium and seeded into the upper chamber with or without Matrigel coated membrane (BD Biosciences, San Jose, CA), while the lower chamber was filled with a fresh medium with 10% FBS. Migrating and Invading cells into the lower surface of the chambers were fixed in paraformaldehyde and stained with DAPI (4′,6-diamidino-2-phenylindole) and reviewed and photographed under a fluorescence microscope at ×200 magnification in three random fields.

### Nude mouse gastric cancer cell xenograft model

Twenty male BALB/c nude mice were purchased from the Guangdong Medical Laboratory Animal Center (Foshan, China). An experimental protocol was approved by the Animal Care and Use Committee (College of Basic Medicine, Jilin University). In brief, BALB/c nude mice were randomly divided into two groups: miRNA controls and miR-31 mimic groups. The dorsal flanks of nude mice were subcutaneously injected with SGC7901/miR-Ctrl and SGC7901/miR-31 (1 × 10^6^ cells per mouse). For the metastasis assay, the cells (2 × 10^6^ cells per mouse) were administered to the mice through the lateral tail vein. Tumor size was examined with vernier caliper once every 3 days after 8 days injection and then calculated to tumor volume by the formula: tumor volume = length × width^2^ × 0.5. Thirty days after tumor cell implantation, all nude mice were sacrificed and tumor xenografts were removed and weighed, moreover, the livers were removed, paraffin-embedded, and subjected to hematoxylin-eosin staining and evaluated by microscope.

### Statistical analysis

All statistical analyses were performed using SPSS version 18.0 (WPSS Ltd, Surrey, UK) and GraphPad Prism 6 software (GraphPad Software, Inc., La Jolla, CA, USA). The results were presented as mean ± s.d. Relative quantification of mRNA and miRNA expression level was calculated with the 2^ΔΔCT^ method. The difference in level of mRNA, miRNA or protein expression between gastric cancer and corresponding normal tissues was evaluated using the nonparametric Mann-Whitney *U*-test. AssociationofmiR-31 andE2F2 expression with clinicopathological parameters was analyzed by using Chi-square test or Fisher's exact probability test. For *in vitro* experiments, Student's *t*-testwas used to analyze difference between two groups. Data with more than two groups were analyzed using a one-way analysis of variance (ANOVA). Association of miR-31 withE2F2 expression was analyzed by using a Pearson correlation test. Kaplan–Meier survival curves were generated to evaluate the correlation of miR-31 and E2F2 expression levels with survival data. All *p*-values were two sided and *p* < 0.05 was considered to be statistically significant.

## SUPPLEMENTARY MATERIALS FIGURE


