# Supplementary material for: Downregulated miR-31 level associates with poor prognosis of gastric cancer and its restoration suppresses tumor cell malignant phenotypes by inhibiting E2F2
Source: Oncotarget. 2016 May 11;7(24):36577–89. doi: 10.18632/oncotarget.9288 (PMC5095022; doi:10.18632/oncotarget.9288)
Supplement: Supplementary file 1 [file oncotarget-07-36577-s001.pdf]

Downregulated miR-31 level associates with poor prognosis of gastric cancer and its restoration suppresses tumor cell malignant phenotypes by inhibiting E2F2

Supplementary Materials

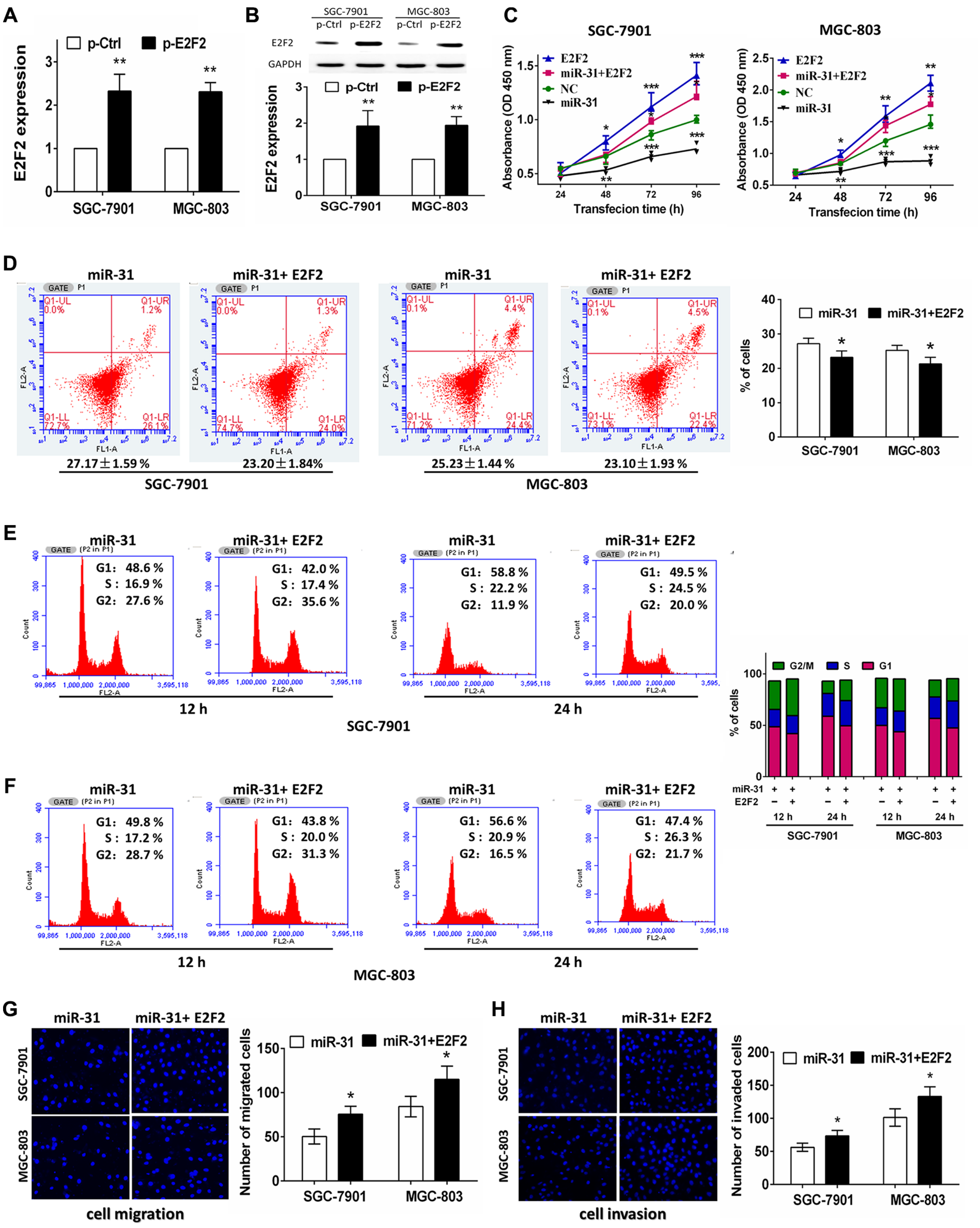

Supplementary Figure S1: Effects of E2F2 restoration on gastric cancer SGC-7901 and MGC-803 cells. Tumor cells were transiently transfected with miR-31 mimics or miR-31 mimics and pWSLV-01-E2F2 plasmid and then subjected to different assays. (**A**) RT-PCR. Level of E2F2 mRNA was assessed by RT-PCR after transfection with pWSLV control or pWSLV-01-E2F2 into gastric cancer cells. (**B**) Western blot. Level of E2F2 protein was assessed after transfection with pWSLV control or pWSLV-01-E2F2 into gastric cancer cells. The relative protein levels were normalized to GAPDH and data were presented as mean ± sd. (**C**) Cell viability CCK8 assay. Cell viability was assessed after 24, 48, 72 and 96 h transfection with miR-31 mimics or miR-31 mimics and pWSLV-01-E2F2 plasmid into gastric cancer cells. Data were presented as mean ± sd of three independent experiments. $*p < 0.05$, $**p < 0.01$ and $***p < 0.001$. (**D**) Apoptosis assay. Level of apoptosis was assessed after 48 h transfection with miR-31 mimics or miR-31 mimics and pWSLV-01-E2F2 plasmid into gastric cancer cells. Data were presented as mean ± sd of three independent experiments with duplicated samples. $*p < 0.05$. (**E–F**) Flow cytometric cell cycle assay. Cell cycle distribution was analyzed additional culture of 12 and 24 h after 48 h transfection with miR-31 mimics or miR-31 mimics and pWSLV-01-E2F2 plasmid into gastric cancer cells. (**G**) Tumor cell migration assay. Fluorescent images (×200) (left) and quantification (right) of migration level of SGC-7901 and MGC-803 cells after 24 h transfection withmiR-31 mimics or miR-31 mimics and pWSLV-01-E2F2 plasmid. Data were presented as mean ± sd, $*p < 0.05$.(**H**) Tumor cell invasion assay. Fluorescent images (left) and quantification (right) of invasion level of SGC-7901 and MGC-803 cells after 24 h transfection with miR-31 mimics or miR-31 mimics and pWSLV-01-E2F2 plasmid.Data were presented as mean ± sd, $*p < 0.05$.
